# Development and validation of a predictive model for the prognosis in aneurysmal subarachnoid hemorrhage

**DOI:** 10.1002/jcla.23542

**Published:** 2020-08-29

**Authors:** Xiang Lai, Wenbo Zhang, Min Ye, Xiaoping Liu, Xingda Luo

**Affiliations:** ^1^ Department of Neurosurgery Meizhou People's Hospital (Huangtang Hospital) Meizhou Hospital Affiliated to Sun Yat‐Sen University Meizhou China

**Keywords:** aneurysmal subarachnoid hemorrhage, discriminant analysis, prognosis, validation

## Abstract

**Background:**

This study was to conduct a predictive model for the prognosis of aneurysmal subarachnoid hemorrhage (aSAH) and validate the clinical data.

**Methods:**

A total of 235 aSAH patients were enrolled in this study, dividing into the favorable or poor prognosis groups based on Modified Rankin Scale (mRS) at 3 months postoperatively. Multivariate analysis was assessed using binary Logistic regression and Fisher discriminant analysis. The receiver operating characteristic (ROC) curve was used to determine the cut‐off value.

**Results:**

Our findings showed that the high Glasgow Coma Scale (GCS) score 24‐hour after surgery reduced the risk of poor prognosis, and the surgical clipping and elevated neutrophil‐lymphocyte ratio (NLR) increased the risk of poor prognosis. The discriminant function was V = 0.881 × GCS score − 0.523 × NLR − 0.422 × therapeutic approach, and V = −0.689 served as a cut‐off value. When V ≥ −0.689, the good prognosis was considered among these patients with aSAH. The correctness for predicting the prognostic outcomes by self‐validation was 85.11%.

**Conclusion:**

This predictive model established by a discriminant analysis is a useful tool for predicting the prognostic outcomes of aSAH patients, which may help clinicians identify patients at high risk for poor prognosis and optimize treatment after surgery.

## Graphical abstract

The ROC curve was established according to V as the test value and mRS score results as the gold standard (AUC = 0.859, SE = .040, 95% CI: 0.781‐0.938) and the critical value of V (cut off) was −0.689. When V ≥ −0.689, the prognosis was considered as good, otherwise as poor

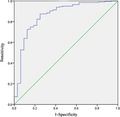

## 1 INTRODUCTION

Aneurysmal subarachnoid hemorrhage (aSAH) is an acute cerebrovascular disease that seriously damages the central nervous system and simultaneously exerts pathophysiological effects on multiple organs of the body, with high mortality and morbidity.^1^, ^2^ Approximately one‐third of the survivors suffer from severe disability and functional dependency.^3^ Rebleeding is a major early complication of aSAH with the incidence of 6%‐23%, which most occurs within 72 hours of onset.^4^, ^5^ Previous studies reported that the mortality was 50%‐60% in the presence of rebleeding.^4^, ^5^, ^6^ To date, craniotomy clipping and endovascular embolization serve as main treatments for aSAH to prevent and treat complications such as rebleeding, vasospasm and hydrocephalus, and to reduce the occurrences of deaths and disabilities.^7^, ^8^, ^9^, ^10^, ^11^ Although improvements in intensive care and treatments in recent years, the prognosis is still a frequent concern for aSAH. To the best of our knowledge, the clinical data indicated that the prognosis of aSAH was poor after surgery. Therefore, prediction of prognostic outcomes is of great value for treatment options and assessment in patients with aSAH.

Several studies have mentioned that biochemical indicators could serve as predictive factors of the prognosis in patients with aSAH, such as lipoprotein‐associated phospholipase A2, high‐sensitivity C‐reactive protein.^12^, ^13^ However, the change of a single indicator may not provide strong and sufficient clinical evidences for clinicians to diagnose the diseases. The predictive models are a derivative tool in statistics which are useful to predict the prognostic outcomes on the basis of the pooled evaluations of physical, laboratory, and radiologic examinations.^14^ High‐quality predictive models can be responsible for guiding clinical decisions and patient counseling, insuring rational allocation of resources to decline the healthcare costs, and improving the designs and analyses of clinical trials.^14^ The optimal care for aSAH patients is applied in clinic, but high mortality cannot be avoided, and the long‐term quality of life among survivors is unsatisfactory postoperatively. The establishment of a productive model may be beneficial in the management of patients with aSAH.

In the current study, we conducted a predictive model for the prognosis of aSAH and validated the clinical data, which may be useful for clinicians to improve the prognostic outcomes of patients after surgery.

## 2 METHODS

### 2.1 Patients

A total of 235 aSAH patients admitted to Meizhou People's Hospital were screened in this study between December 2016 and December 2018. All cases were consecutively recruited in the retrospective investigation that were divided into the favorable (n = 201) or poor (n = 34) prognosis groups based on Modified Rankin Scale (mRS) at 3 months postoperatively.^15^ In this study, patients at score 4‐6 manifested poor prognosis, while patients at score 0‐3 showed favorable prognosis. The clinical indicators were noted including gender, age, body mass index (BMI), drinking, smoking, diabetes, hypertension, operation history, Hunt‐Hess grade, surgical techniques, surgical timing, initially ruptured aneurysm bleeding, aneurysm numbers and sizes, Glasgow Coma Scale (GCS) score, biochemical indexes, and prognostic outcomes. This study was approved by the Institutional Review Board (IRB) of Meizhou People's Hospital (approval number: No. 2016‐A‐39).

Inclusion criteria: (a) 18‐80 years old; (b) clearly diagnosed as aSAH according to guidelines for the diagnosis and treatment of Chinese subarachnoid hemorrhage; (c) CT scan of the head confirmed aSAH; (d) patients with first rupture of aneurysm by CT angiography (CTA) and digital subtraction angiography (DSA); (e) admission within 24 hours of the initial symptom onset; (f) hospitalization ≥3 days in neurosurgery.

Excluded criteria: (a) aSAH patients caused by non‐aneurysmal causes such as arteriovenous malformation or arteriovenous fistula; (b) patients with histories of acute or chronic infections, autoimmune diseases, recent cardiovascular and cerebrovascular diseases, and other systemic diseases including malignant neoplasms, uremia, chronic heart disease, and chronic pulmonary disease; (c) previous use of anticoagulant/antiplatelet drugs; (d) patients with histories of acute craniocerebral trauma, brain tumor or stroke with neurological dysfunction; (e) incomplete clinical data; (f) patients with preoperative rebleeding of aneurysms and surgical treatment failure; (g) severe liver and kidney dysfunction; (h) refuse or lost to follow‐up; (i) patients who have taken other experimental drugs or ongoing clinical studies within 1 month.

### 2.2 Laboratory examination

The blood biochemical indexes were tested using the automatic biochemical analyzer. The clinical indicators consisted of high‐density lipoprotein (HDL), low‐density lipoprotein (LDL), heart rate (HR), neutrophil‐lymphocyte ratio (NLR), platelet‐lymphocyte ratio (PLR), white blood cell (WBC) count, platelet (PLT) count, neutrophil count (NEUT), lymphocyte count (LC), glutamic oxaloacetic transaminase (AST), albumin and fasting blood glucose (FBG).

### 2.3 GCS score

Glasgow Coma Scale score mainly includes Motor response (M), Verbal response (V), and eye‐opening (E). The degree of coma is evaluated based on the sum of M, V, and E scores. A score above 14 was considered as normal, a score below 7 was coma, and GCS score ≤3 was indicated brain death or poor prognosis.

### 2.4 Statistical analysis

Statistical analysis was performed using SPSS 23.0 (SPSS, Inc, Chicago, IL). Measuring data were presented as the mean ± standard $(\bar{x}\pm \text{s)}$ and analyzed by ANOVA. Counting data were presented as n (%) with χ^2^ or Fisher tests. The single‐factor analysis of the prognosis at 3 months postoperatively was carried out by *t*, Kruskal‐Wallis or χ^2^ tests. Multivariate analysis was assessed using binary Logistic regression and Fisher discriminant analysis. The receiver operating characteristic (ROC) curve was used to determine the cut‐off value. *P* < .05 was considered statistically significant.

## 3 RESULTS

### 3.1 The baseline data of patients with aSAH

**Figure 1:**
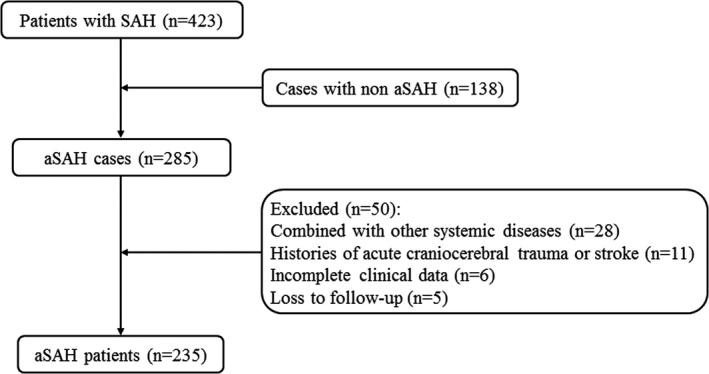
ROC curve of the prognosis in patients with aASH

The process of patient selection was shown in Figure 1. Totally, 235 patients with aSAH were included in this study containing 93 males (39.60%) and 142 females (60.40%), with the mean age of (58.10 ± 9.97) years and the mean BMI of (23.08 ± 2.20). There were no statistical differences in gender (*χ^2^* = 0.04, *P* = .836), age (*t* = −1.01, *P* = .311), BMI (*t* = 1.29, *P* = .199), drinking (*χ^2^* = 0.016, *P* = .899), smoking (*P* = .378), diabetes (*P* = .068), hypertension (*χ^2^* = 1.23, *P* = .268), operation history (*P* = .610), surgical timing (*P* = .345), initially ruptured aneurysm bleeding (*P* = .855), aneurysm numbers (*χ^2^* = 1.956, *P* = .162), and sizes (*P* = .153) between favorable and poor prognosis groups. Significant differences in Hunt‐Hess grade (*χ^2^* = 63.58, *P* < .001) and surgical techniques (*χ^2^* = 14.31, *P* < .001) were showed between the two groups (Table 1).

**Table 1:** The characteristics of various prognostic outcomes in patients with aSAH

Variables	Favorable prognosis	Poor prognosis	Total	χ^2^/t	*P*
Gender, n (%)					
Male	79 (84.90)	14 (15.10)	93 (39.60)	0.04	.836
Female	122 (85.90)	20 (14.10)	142 (60.40)		
Age, years,$\bar{x}\pm s$	57.83 ± 9.87	59.71 ± 10.53	58.10 ± 9.97	−1.01	.311
BMI, Kg/m^2^,$\bar{x}\pm s$	23.16 ± 2.19	22.64 ± 2.23	23.08 ± 2.20	1.29	.199
Drinking, n (%)					
No	126 (84.60)	23 (15.40)	149 (78.40)	0.016	.899
Yes	35 (85.40)	6 (14.60)	41 (21.60)		
Smoking^a^, n (%)					
No	131 (83.40)	26 (16.60)	157 (85.80)		.378
Yes	24 (92.30)	2 (7.70)	26 (14.20)		
Diabetes^a^, n (%)					
No	143 (85.60)	24 (14.40)	167 (91.80)		.068
Yes	10 (66.70)	5 (33.30)	15 (8.20)		
Hypertension, n (%)					
No	73 (82.00)	16 (18.00)	89 (44.10)	1.23	.268
Yes	99 (87.60)	14 (12.4)	113 (55.90)		
Operation history, n (%)					
No	141 (83.90)	27 (16.10)	168 (95.50)		.610
Yes	8 (100.00)	0 (0.00)	8 (4.50)		
Hunt‐Hess grade, n (%)					
I‐III	178 (94.70)	10 (5.30)	188 (80.00)	63.58	<.001
IV‐V	23 (48.90)	24 (51.10)	47 (20.00)		
Surgical techniques, n (%)					
Endovascular therapy	114 (94.20)	7 (5.80)	121 (51.50)	14.31	<.001
Surgical clipping	87 (77.00)	26 (23.00)	113 (48.10)		
Surgical timing^a^, n (%)					
Early stage	183 (86.30)	29 (13.70)	212 (90.20)		.345
Delay	18 (78.30)	5 (21.70)	23 (9.80)		
Initially ruptured aneurysm bleeding^a^, n (%)					
No	1 (100.00)	0 (0.00)	1 (0.40)		.855
Yes	199 (85.40)	34 (14.60)	233 (99.60)		
Aneurysm, n (%)					
Single	179 (86.50)	28 (13.50)	207 (88.20)	1.956	.162
Multiple	19 (76.00)	6 (24.00)	25 (10.89)		
Aneurysm size^a^, n (%)					
<13 mm	198 (86.10)	32 (13.90)	230 (97.90)		.153
≥13 mm	3 (60.00)	2 (40.00)	5 (2.10)		

^a^ Using Fisher test.

### 3.2 Single‐factor regression analysis for the prognosis at 3 months postoperatively

The GCS score (*H* = 54.46, *P* < .001) and albumin (*t* = 2.70, *P* = .008) in the favorable prognosis group was higher than that in the poor prognosis group, with statistical differences. The levels of NLR (*t*′ = −3.68, *P* = .001), PLR (*t*′ = −2.23, *P* < .001), HR (*t*′ = −2.70, *P* = .010), WBC count (*t*′ = −3.61, *P* < .001), NEUT (*t* = 3.79, *P* < .001), AST (*t*′ = −2.45, *P* = .015), and FBG (*t*′ = −2.32, *P* = .028) in the poor prognosis group were higher than the favorable group. We also found that there were no statistical differences in rebleeding or death 24 hours after onset (*P* = .054), HDL (*z* = 1.39, *P* = .326), LDL (*t* = 1.35, *P* = .177), PLT count (*t* = 0.20, *P* = .838), and LC (*z* = 1.53, *P* = .295) between the two groups (Table 2).

**Table 2:** The characteristics of aSAH patients between the two groups at 3 mo postoperatively

Variables	Favorable prognosis	Poor prognosis	Total		*P*
GCS score, n (%)					
3‐8	22 (10.95)	21 (61.80)	43 (18.40)	*H* = 54.46	<.001
9‐12	10 (4.98)	4 (11.80)	14 (6.00)		
13‐15	168 (83.58)	9 (26.50)	177 (75.60)		
Rebleeding or death 24 h after onset^a^, n (%)					
None	196 (99.49)	32 (94.12)	228 (98.70)		.054
Rebleeding or death	1 (0.51)	2 (5.88)	3 (1.30)		
HDL^b^, mmol/L, M(P_25_, P_75_)	1.35 (1.15, 1.59)	1.30 (1.10, 1.77)	1.34 (1.14, 1.62)	*z* = 1.39	.326
LDL, mmol/L, $(\bar{x}\pm \text{s)}$/M(P_25_, P_75_)	3.14 ± 0.89	2.90 ± 0.99	3.11 (2.55,3.60)	*t* = 1.35	.177
NLR^c^,$\bar{x}\pm s$	7.04 (5.10, 11.62)	11.20 (7.17, 17.44)	7.37 (5.54, 12.31)	*t*′ = −3.68	.001
PLR^c^, M(P_25_, P_75_)	157.39 (111.90, 203.56)	180.80 (122.00, 252.88)	160.79 (112.30, 206.81)	*t*′ = −2.23	.033
HR^c^, beats/min,$\bar{x}\pm s$	81.51 ± 9.48	89.50 ± 16.79	82.67 ± 11.17	*t*′ = −2.70	.010
WBC count,$\bar{x}\pm s$	13.89 ± 4.64	17.04 ± 4.74	14.34 ± 4.77	*t* = −3.61	<.001
PLT count,$\bar{x}\pm s$	229.73 ± 56.97	227.48 ± 67.09	229.41 ± 58.35	*t* = 0.20	.838
NEUT,$\bar{x}\pm s$	11.49 ± 4.48	14.65 ± 4.27	11.93 ± 4.58	*t* = 3.79	<.001
LC^b^, M(P_25_, P_75_)	1.50 (1.10, 2.00)	1.10 (0.90, 1.90)	1.40 (1.10, 2.00)	*z* = 1.53	.295
AST, U/L,$\bar{x}\pm s$	26.61 ± 12.84	32.79 ± 15.09	27.50 ± 13.33	*t* = −2.45	.015
Albumin, g/L,$\bar{x}\pm s$	42.85 ± 4.35	40.50 ± 6.06	42.51 ± 4.70	*t* = 2.70	.008
FBG^c^, mmol/L,$\bar{x}\pm s$	7.02 ± 2.53	8.69 ± 3.39	7.22 ± 2.69	*t*′ = −2.32	.028

Abbreviations: AST, glutamic oxaloacetic transaminase; FBG, fasting blood‐glucose; GCS, Glasgow Coma Scale; HDL, high‐density lipoprotein; HR, heart rate; LC, lymphocyte count; LDL, low‐density lipoprotein; NEUT, neutrophil count; NLR, neutrophil‐lymphocyte ratio; PLR, platelet‐lymphocyte ratio; PLT, platelet; WBC, white blood cell.

^a^ Using Fisher test;

^b^ Using Mann‐Whitney *U* test;

^c^ Using *t* test.

### 3.3 Multivariate Logistic regression analysis for the prognosis at 3 months postoperatively

In Table 3, the stepwise regression analysis was used to analyze the influence factors of the prognosis at 3 months postoperatively in patients with aSAH. The findings showed that the higher the GCS score 24‐hour after surgery, the lower the risk of poor prognosis (OR = 0.34, 95% CI: 0.18‐0.63, *P* = .001). The risk of poor prognosis in the surgical clipping was higher than that in the endovascular therapy (OR = 3.34, 95% CI: 1.02‐10.99, *P* = .033). In addition, the higher the NLR, the higher the risk of poor prognosis (OR = 1.13, 95% CI: 1.03‐1.24, *P* = .008).

**Table 3:** Multivariate Logistic regression analysis for the prognosis at 3 mo postoperatively

Variables	OR (95% CI)	B	*P*
GCS scoring at 24 h after surgery	0.34 (0.18‐0.63)	−1.09	.001
Surgical techniques			
Endovascular therapy	—		
Surgical clipping	3.34 (1.02‐10.99)	1.21	.033
NLR	1.13 (1.03‐1.24)	0.12	.008

Abbreviations: CI, confidence interval; GCS, Glasgow Coma Scale; NLR, neutrophil‐lymphocyte ratio; OR, odds ratio.

### 3.4 The predictive model for the prognosis at 3 months postoperatively

The GCS score 24‐hour after surgery, surgical techniques, and NLR was as independent variables, the prognosis at 3 months postoperatively was as the grouping variable, and Fisher discriminant analysis was carried out. Then, the discriminative equation was as follows: V = 0.881 × GCS score − 0.523 × NLR − 0.422 × surgical techniques. The value of Wilks'Lambda was 0.712 (*χ^2^* = 77.36, *P* < .001), with significant differences.

**Figure 2:**
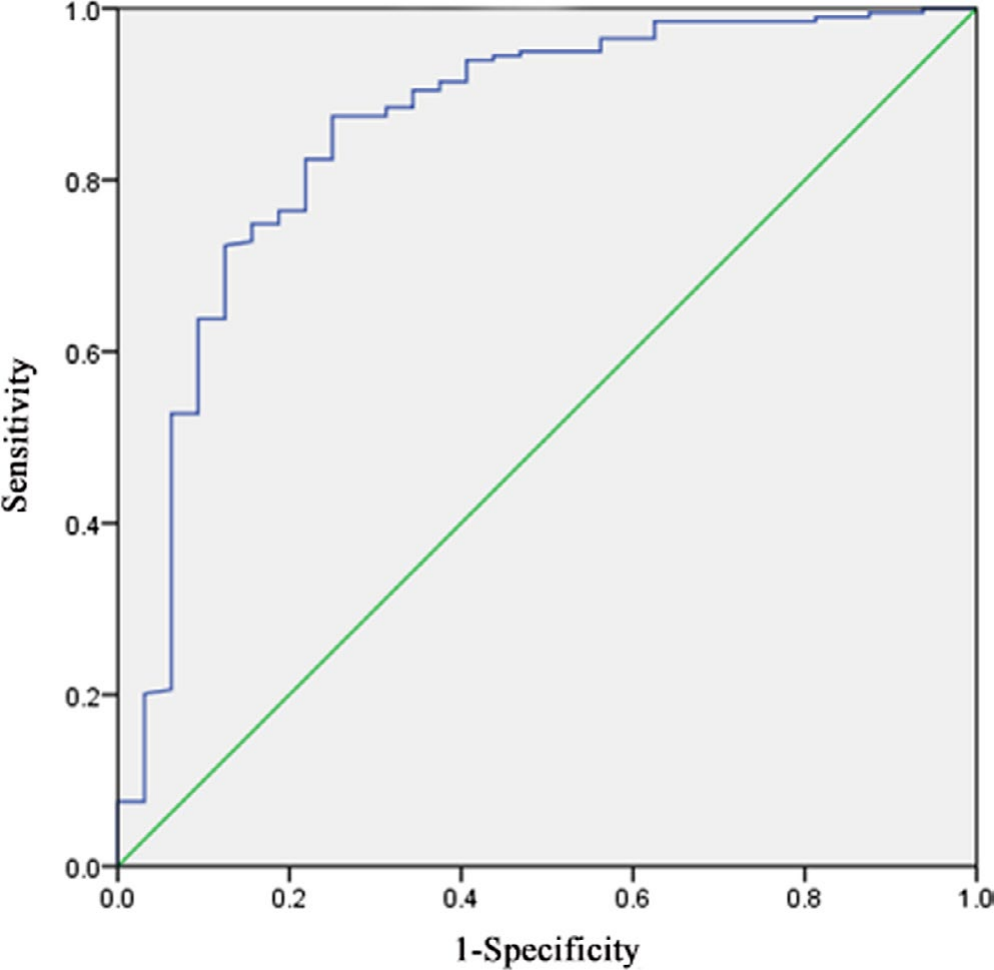
ROC curve of the prognosis in patients with aASH

The GCS score (score 3‐8 as 1, score 9‐12 as 2 and 13‐15 as 3) and surgical techniques (endovascular therapy as 1 and surgical clipping as 2) were assigned in this study. The V‐value was calculated based on the discriminative equation. The ROC curve (Figure 2) was established according to V as the test value and mRS score results as the gold standard (AUC = 0.859, SE = .040, 95% CI: 0.781‐0.938) and the critical value of V (cut off) was −0.689. When V ≥ −0.689, the prognosis was considered as good, otherwise as poor.

The self‐validation method was used to assess the efficacy of the predictive model in Table 4. The V‐value of 182 patients with aSAH was >−0.689, indicating favorable prognosis, while 53 cases were poor prognosis (V<−0.689). The results showed that 86.57% of patients were accurately recognized with a good prognosis via the predictive model in the favorable prognosis group. 76.47% of subjects were correctly judged as having a bad prognosis in the poor prognosis group. The accuracy of this predictive model obtained by the discriminant analysis was 85.11%. It is indicated that the predictive effect of the model was relatively reasonable.

**Table 4:** The self‐validation of the predictive model

Predictive outcomes	Favorable prognosis (score 0‐3)	Poor prognosis (score 4‐6)
Favorable prognosis	86.57% (174/201)	23.53% (8/34)
Poor prognosis	13.43% (27/201)	76.47% (26/34)

## 4 DISCUSSION

Aneurysmal subarachnoid hemorrhage is a serious disease that threatens human health. Previous studies reported that >30% of mortality was related to the existence of aSAH, and merely about 30% of aSAH patients could return to independent living.^16^ Approximately 10%‐25% of acute aSAH patients die after bleeding or before arrival at the hospital.^17^ Thus, it is of great value to pay attention to the prognostic outcomes of aSAH patients to improve their quality of life and survival. In this study, we established a predictive model to assess the prognosis of aSAH using a discriminant analysis, and to conduct an internal validation to identify the effectiveness of this model. Totally, 235 aSAH patients were screened and observed the recovery 3 months after surgery. Patients at score 4‐6 manifested poor prognosis, while patients at score 0‐3 showed favorable prognosis. The prognostic parameters including the GCS score 24‐hour after surgery, surgical clipping, and NLR were used to establish a discriminant function, with an accuracy of 85.11%. Our findings showed that high GCS score 24‐hour after surgery was a protective factor for the risk of poor prognosis, and the surgical clipping and NLR were risk factors for the occurrence of poor prognosis in patients with aSAH.

Studies have shown that the discriminant analysis has been clinically used to select significant indicators.^18^, ^19^, ^20^ In this work, we first applied this analysis to establish a predictive model to screen the major factors of prognosis in patients with aSAH, which may be availably applied in clinic. Our findings from multivariate Logistic analysis showed that the high GCS score 24‐hour after surgery could reduce the risk of poor prognosis, and the surgical clipping and elevated NLR could increase the risk of poor prognosis. The discriminant function was conducted with these significant variables, V = 0.881 × GCS score − 0.523 × NLR − 0.422 × surgical techniques, and V = −0.689 served as a cut‐off value. When V ≥ −0.689, the good prognosis was considered among these patients with aSAH. We further demonstrated that the accuracy of the discriminant analysis for predicting the prognostic outcomes was 85.11%, of which 96.57% in the feature of favorable prognosis and 76.47% of poor prognosis. It was indicated that the predictive effectiveness of this model was relatively reasonable, which may be generalized to the clinic for the prediction of the prognostic outcomes among aSAH cases, improving the living quality and survival condition.

Previous studies have demonstrated that NLR was associated with the outcomes and could predict the courses of different medical conditions, such as ischemic stroke,^21^, ^22^ cerebral hemorrhage,^23^, ^24^ and major cardiac events.^25^, ^26^ In our study, we found that NLR was associated with the prognostic outcomes of aSAH patients. The high level of NLR could increase the risk of poor prognosis at 3 months postoperatively. One mechanism by which neutrophils can contribute to unfavorable outcomes following aSAH is the synthesis and secretion of matrix metalloproteinase. These are enzymes able to degrade any component of the extracellular matrix and play role in brain‐barrier damage and development of secondary brain injury.^27^ In addition, the mechanism of NLR‐induced bleeding may be contributed by multifactorial pathophysiology, such as inflammatory reaction, immune dysfunction, and the production of reactive oxygen species (ROS).^5^, ^28^, ^29^, ^30^, ^31^, ^32^ aSAH can induce the leukocytosis and increased neutrophils by stimulating the systemic cellular responses that can cause the brain injury and delayed cerebral ischemia.^33^, ^34^ Early studies showed that the growth and rupture of cerebral aneurysms were promoted via the leukocyte infiltration and inflammatory reaction in the wall of aneurysms.^35^, ^36^, ^37^, ^38^ The results of histopathology found that the thinned wall of aneurysms was related to leukocytosis.^39^ Furthermore, Sheheryar et al mentioned that elevated NLR at admission could predict higher inpatient mortality among aSAH patients.^40^ Giede‐Jeppe et al^29^ reported NLR as an independent factor for unfavorable functional outcome in aSAH. These were consistent with our findings, indicating the high level of NLR may increase the risk of poor prognosis in patients with aSAH.

In addition, we also discovered that the risk of poor prognosis in the surgical clipping was higher in comparison with the endovascular therapy. The surgical clipping is a gold standard treatment in recent decades, and Yasargil applied the microsurgical techniques to neurosurgery to improve the surgical approach to intracranial aneurysms.^41^ Endovascular therapy was initially based on the use of inflatable balloons in the aneurysmal cavity in 1970s.^42^ Endovascular coiling has been served as a succedaneous method for treating the ruptured or unruptured intracranial aneurysms after approval of Food and Drug Administration (FDA) in 1995. Previous studies reported the better effectiveness of endovascular therapy in quality of life and survival in comparison with surgical clipping.^43^, ^44^ Compared with those treated with surgical clipping, the mortality and disability of ruptured intracranial patients receiving 1‐year endovascular therapy were lower, with a reduced absolute risk of 7.4%.^44^ These were similar with our results, suggesting the surgical clipping for aSAH may increase the risk of poor prognosis.

A strength of this study is that we established a predictive model for the prognosis of aSAH, and validated with the internal data. The high GCS score 24‐hour after surgery was a protective factor, and the surgical clipping and NLR were risk factors for the occurrence of poor prognosis in aSAH patients. The establishment of our discriminant function had a good performance for predicting the prognostic outcomes in aSAH patients. There were some limitations in our study. First, the sample size may reduce the statistical power. Second, only a small number of enrolled patients developed poor prognosis, which may affect the statistical outcomes. Third, due to the possible influence of treatments, the longitudinal assessment of laboratory indices over time was not carried out. Additionally, the predictive value of this model was evaluated base on an internal data, lacking of the external validation. These should be cautious in interpreting the results. Hence, future studies should further validate the results of the present study.

## 5 CONCLUSION

We established a predictive model to assess the prognosis of aSAH using a discriminant analysis, and to conduct an internal validation to identify the effectiveness of this model. Our results revealed that the correctness for predicting the favorable prognosis was 85.67%, as well as for predicting the poor prognosis was 76.47%. The accuracy obtained by discriminant analysis was 85.11%, indicating that the effectiveness of this predictive model was relatively reasonable. These findings obtained from our study may help clinicians identify patients at high risk for poor prognosis and optimize treatment after surgery.
